# Intra-Articular Injection of Human Umbilical Cord-Derived Mesenchymal Stromal Cells Reduces Radiographic Osteoarthritis in an Ovine Model

**DOI:** 10.1177/19476035241287832

**Published:** 2024-11-03

**Authors:** Jade Perry, Claire Mennan, Paul Cool, Helen S. McCarthy, Karin Newell, Timothy Hopkins, Charlotte Hulme, Karina T. Wright, Frances M.D. Henson, Sally Roberts

**Affiliations:** 1 The Robert Jones and Agnes Hunt Orthopaedic Hospital NHS Foundation Trust, Oswestry, UK; 2 Centre of Regenerative Medicine Research, The School of Pharmacy and Bioengineering, Keele University, Staffordshire, UK; 3 The Tissue Engineering & Regenerative Therapies Centre, Versus Arthritis, Chesterfield, UK; 4 Department of Veterinary Medicine, University of Cambridge, Cambridge, UK; 5 Centre for Predictive In Vitro Models, Queen Mary University of London, London, UK; 6 Centre for Bioengineering, School of Engineering and Materials Science, Queen Mary University of London, London, UK; 7 Department of Surgery, University of Cambridge, Cambridge, UK

**Keywords:** cell therapy, allogeneic, mesenchymal stem cells, cells, osteoarthritis, diagnosis, preclinical models

## Abstract

**Objective:**

To determine if mesenchymal stromal cells (MSCs) derived from human umbilical cords (hUC) could reduce degeneration developing when injected into the knee of a large animal model of osteoarthritis (OA).

**Design:**

Ten million culture-expanded UC-MSCs (pooled from 3 human donors) were injected in 50 μL of tissue culture medium into the left stifle joints of 7 sheep whose medial meniscus was transected 4 weeks previously. Seven other sheep had only 50 μL of medium injected as the no treatment “control” group. After 8 weeks the sheep underwent euthanasia, the joints were excised and examined macroscopically, via x-ray and magnetic resonance imaging (MRI), both via histology for degenerative and inflammatory changes and immunohistochemically to identify any human cells within the joint tissues. Activity monitoring both before meniscus transection and euthanasia was also undertaken.

**Results:**

There was a significant reduction in the Kellgren–Lawrence x-ray score for joints injected with hUC-MSCs compared with the control joints. Likewise, macroscopic, MRI, synovitis and OARSI histology scores were all lower (better) in the joints injected with hUC-MSCs than in the control arm, but not significantly. Activity levels and synovitis scores were similar in both groups of animals.

**Conclusions:**

hUC-MSCs appear to modify and reduce the development of osteoarthritic changes in the ovine stifle joint after meniscal destabilization, an injury which commonly leads to OA in humans. These results are encouraging for the potential benefit of culture expanded UC-MSCs as an allogeneic cell therapy in patients who may have early OA following a meniscal injury of the knee.

## Introduction

Despite osteoarthritis (OA), particularly in the knee, being a major global health concern,^1^ there are few effective disease modifying treatments to change its natural history and progression. A biological approach using cell therapy offers the promise of an early and perhaps permanent solution for treating OA, particularly if it could be simplified and made even more cost effective than the current option of autologous chondrocyte implantation (ACI). With this in mind, various cell therapies are currently being trialed, including intra-articular injections using mesenchymal stromal cell (MSCs), with the most common sources for clinical use being bone marrow-derived (BM)-MSCs^2^ and adipose-derived (AD)-MSCs.^3-8^ We and others have investigated MSCs derived from human umbilical cords (hUC-MSCs), which have a similar trilineage differentiation capability, immunomodulatory ability and CD-immunoprofiles compared with hBM-MSCs, whilst also having greater proliferative capacity.^9,10^ While the exact mode of action (MoA) of MSCs remains unknown, increasing evidence suggests that MSCs function through trophic effects on endogenous cell populations, with the production of several soluble factors, such as growth factors and cytokines, immunomodulatory and anti-inflammatory molecules,^11^ as well as the secretion of extracellular vesicles (EVs) which contain many of these factors.^12^ Furthermore, through the prevention of T-lymphocyte maturation and reduction in macrophage activation, MSCs appear capable of minimizing local inflammatory responses in arthritic joints.^13^

An allogeneic MSC product offers several advantages over an autologous one; for example, the patient requires only a single procedure, as no cell harvest is required. This prevents short-term donor site morbidity, saves costs and allows easier logistics in delivering the treatment. In addition, there are lower production and commercial manufacturing costs for allogeneic cell products than autologous ones as several treatment batches can be prepared in one manufacturing run rather than a single treatment as with an autologous product. Furthermore, autologous MSCs derived from a patient with OA are likely to have reduced *in vitro* proliferation and differentiation potential compared with cells derived from tissues earlier in development.^14^ Human UC-MSCs therefore appear to have exciting potential as a source of allogeneic cells in the prevention/treatment of OA.

Previously, we have studied the effect of intra-articular injected hUC-MSCs *in vivo* in 2 small preclinical models. One was in a murine model of joint surface injury (JSI)^15^ which in some ways resembles the clinical situation (i.e., an isolated cartilage defect) that ACI was designed to treat, rather than OA *per se*. The other was a murine model of established severe OA, the partial medial meniscectomy (PMM) model.^16^ In the JSI model, mice treated with hUC-MSCs demonstrated significantly improved repair tissue formation in the site of injury, compared with that seen in the no-cell (control) group of mice.^15^ However, these differential findings were not present in mice with end-stage OA in the PMM model.^16^ It is of note that in both of these xenogeneic models, the implanted hUC-MSCs did not appear to elicit any inflammatory reaction.

Following these murine models, the next logical step was to apply hUC-MSCs to a large animal (ovine) model of early to moderate OA. Hence, this study was undertaken to apply an intra-articular injection of hUC-MSCs into ovine knees (stifle joints), which had undergone a medial meniscectomy and to monitor the progression of OA using several different methods.

## Materials and Methods

### Human Samples

All human umbilical cords were collected after maternal donors had provided written informed consent, with favorable ethical approval being given by the National Research Ethics Service (10/H1013/62). Umbilical cords (*n* = 3) were obtained following natural births, from healthy mothers aged 23 to 35 years, and processed within 24 hours of delivery as previously described.^9^ Human UC-MSCs were isolated from the tissue enzymatically and culture-expanded via a hybrid process.^9,15^ Initially, hUC-MSCs were cultured for a single passage using standard tissue culture techniques in complete culture medium, Dulbecco’s Modified Eagle’s Medium (DMEM/F12, Life Sciences, Paisley, UK) containing 1% (*v/v*) penicillin and streptomycin (P/S, Life Sciences, Paisley, UK) and 10% (*v/v*) fetal calf serum (FCS, Life Sciences, Paisley, UK)^9,15^ for 12 to 20 days. They were then harvested via trypsinization at 70% to 80% confluency and 5 million cells seeded into the Quantum^®^ bioreactor (Terumo BCT Inc, Lakewood, CO) in complete culture medium for 6 to 11 days before being harvested and characterized or stored frozen in liquid nitrogen until application.^9^

### Human UC-MSC Characterization

Human UC-MSC populations were assessed for the presence of the International Society for Cellular Therapy (ISCT) MSC markers^9^: CD19, CD34, CD45, Human Leukocyte Antigen (HLA)-DR (−ve), CD73, CD90, CD105 (+ve), as well as some chondrogenic/MSC markers: CD271, Receptor Tyrosine Kinase-like Orphan Receptor 2 (ROR2), Fibroblast Growth Factor Receptor 3 (FGFR3), CD151, CD39, CD44, CD49, CD163, CD166 and immunomodulatory markers: CD106 and CD317, by flow cytometry with appropriate isotype-matched IgG negative controls as previously described.^9,15^

### Animals

All animal experimental protocols were carried out in accordance with the Animal Scientific Procedures Act (1986) and are reported in compliance with the Animal Research: Reporting of In Vivo Experiments (ARRIVE) guidelines. This study was approved by the UK Home Office and the Animal Welfare and Ethical Review Board at Cambridge University. Female, skeletally mature Welsh Mountain sheep (aged 3-4 years), underwent a medial meniscus transection surgery in the left stifle joint, a model that induces OA over 12 weeks,^17^ as previously described.^18^

### Animal Anesthesia, Preparation, and Surgical Techniques

Sheep (*n* = 14) were anesthetized with an intravenous injection of 0.25 mg/mg alfaxalone and anesthesia maintained via inhalation of isofluorane. The surgical procedure was performed identically for all subjects and performed under strict sterile conditions by a single surgeon (F.M.D.H.). Prior to surgery each stifle joint was physically examined to ensure there were no abnormalities; if any gross pathology or instability was present, the animal was excluded from participating in the study.

If suitable, the sheep was placed in dorsal recumbency and the (left) medial femorotibial joint opened from the medial aspect, cranial to the collateral ligament, leaving the medial collateral ligament intact. The medial meniscus was identified and the superior surface transected, using a No. 11 scalpel blade as previously described.^17,18^ The tibial plateau was protected via a rounded soft polypropylene spacer inserted via an incision at the inferior surface junction to allow access. The incision was then closed using vertical mattress sutures (0 Vicryl, Ethicon) through the joint capsule and Vicryl absorbable sutures (3-0, Ethicon) to close the skin. For the first 72 hours following surgery, post-operative analgesia (intramuscular 4 mg/kg carprofen) was provided once daily to provide pain relief. No animals received any immobilization techniques, splints or casts. All animals were allowed to fully weight bear following surgery, initially being kept in a small pen for 48 hours for monitoring and to reduce ambulation, before being maintained for a further 5 days in a large indoor pen, after which they went outdoors to pasture in a field. Sheep were monitored for 4 weeks post-surgery for behavioral changes or poor wound healing.

### Cell Application

All animals in this study were randomly allocated to their treatment groups—either cells + delivery vehicle (hUC-MSCs in DMEM/F12) or delivery vehicle alone (DMEM/F12). On the day prior to surgery a member of the technical staff (not part of the research team) randomly chose which animals were to be used the following day and in what order animals would be operated on and subsequently treated. Treatments were given by intra-articular injection into the medial femoro-tibial joint of the operated joint under aseptic conditions 4 weeks post-surgery as described below.

**Figure 1.**
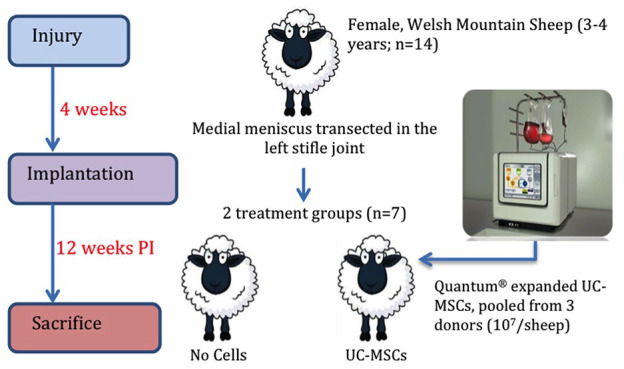
Experimental design. Quantum expanded human UC-MSCs (10^7^ cells in total, pooled from 3 individual donors) or DMEM/F12 alone (control group) were injected into the left stifle joint of Welsh mountain sheep 4 weeks post-medial meniscus transection surgery. Sheep were culled 12 weeks post-injury (PI).

Prior to treatment, the Quantum^®^ expanded hUC-MSCs from 3 individual human donors were thawed, washed in DMEM/F12 and pooled to ensure a consistent cell product was injected into all 7 animals. All hUC-MSCs were found to be negative (<2%) for CD19, CD34, CD45, Human Leukocyte Antigen (HLA)-DR and positive (>95%) for CD73, CD90, CD105. Ten million cells in 50 μL of DMEM/F12 were injected intra-articularly in the treatment group (*n* = 7; **Fig. 1**). No cells were administered to those in the control group (*n* = 7), but 50 μL of DMEM/F12 was injected representing the vehicle control. Following surgery, all animals were housed again as a single flock and all further analyses performed in a random order, for example, gait analysis.

Two sheep in the treatment group demonstrated lameness 1-day post-injection, so received anti-inflammatory medication (Carprofen) for 48 hours, as per the Home Office guidelines, after which the lameness had resolved. Sheep were sacrificed, using an overdose of injectable anesthetic, 8 weeks post-injection of cells or vehicle alone. Joints were retrieved, assessed for their macroscopic appearance, imaged with x-rays and magnetic resonance imaging (MRI) and then fixed in formalin (4% formaldehyde v/v) for histological analysis.

### Activity Monitoring

Dynamic incapacitance (weight bearing was measured using an “Accugait” force plate; AMTI, MA) prior to surgical treatment and ~12 weeks post-surgical treatment, prior to euthanasia.^19^ Dynamic weight bearing was calculated as a percentage of the weight bearing for each sheep post-treatment compared with that prior to the injury (K.N., F.M.D.H.).

### Post-mortem Joint Evaluation

At post-mortem, the gross morphology of the operated knee joints was scored by 2 blinded observers (K.N., F.M.D.H.) as previously described.^20^ In brief, the stifle joints were divided into 4 compartments: the medial femoral condyle (MFC), lateral femoral condyle (LFC), medial tibial plateau (MTP), and lateral tibial plateau (LTP). At each anatomical site, macroscopic scoring of gross articular damage (0-16) and osteophyte development (0-12) was performed. Macroscopic synovial changes were also assessed (0-5).^20^ Hence total scores could range from 0 to 33, where a high score indicated greatest degeneration to the joint.

### Radiographic Evaluation

Following euthanasia, cranio-caudal radiographs were obtained of the operated limbs using a Cloud DR scanner (BCF Technologies). Two blinded observers (J.P., P.C.) scored the x-rays with the Kellgren–Lawrence (KL) scoring system,^21^ ranging from 0 to 4, where a high KL score indicates increased osteoarthritic damage to the joint, whilst a low score indicates a normal healthy joint.

### Magnetic Resonance Imaging

Joints were also imaged *ex vivo*, with a 0.25-T MRI scanner (Esaote). The imaging protocol used the following sequence: T1 echo train = 1, TR = 0.0 ms, TE = 26.0 ms, slice thickness = 2.5 mm, dimension size = 2.5 × 2.5 mm^2^, matrix size = 256 × 256; T2 echo train = 8, TR = 0.0 ms, TE = 120.0 ms, slice thickness = 4.0 mm, dimension size = 4.4 × 4.4 mm^2^, matrix size = 512 × 512 and 3D T2-weighted hybrid contrast-enhanced (Hyce) echo train = 1, TR = 0.0 ms, TE = 21.1 ms, slice thickness = 2.5 mm, dimension size = 2.5 × 2.5 mm^2^, matrix size 512 × 512.

The sheep Magnetic resonance Osteoarthritis Knee Score (sMOAKS)^22^ was used to evaluate joint degeneration in the ovine knee joints and the images were scored by 2 blinded observers (J.P., P.C.) from 0 to 234.^22^ The joint was divided into the 3 main compartments: the femoro-tibial medial joint (FTMJ) and femoro-tibial lateral joint (FTLJ) and the patellofemoral joint (PFJ). In all compartments, articular cartilage loss (0-72), bone marrow lesions (BMLs) and cysts (0-108) and osteophyte formation (0-48) were measured, alongside Hoffa’s synovitis (0-3) and effusion (0-3). In all cases a high score indicates increased osteoarthritic damage to the joint, whilst a low score indicates a normal healthy joint.

The thickness and total volume of the femoral cartilage were also measured on the MR scans of the operated ovine knee joints using pyKNEER, as previously described.^23^ High-resolution 3-Tesla MR sheep scans from a normal ovine knee joint were used as the reference template. In brief, the automatic image analysis workflow preprocesses, segments and analyses femoral knee cartilage from MR images. T1- and T2-weighted images were used for the cartilage segmentation and manually checked by 2 authors (J.P., P.C.).

### Histological Evaluation

Following imaging, the 4 main joint quadrants (MFC, LFC, MTP, and LTP) and synovium from the operated knee joints were isolated for histological processing. After fixation in 10% neutral buffered formalin, samples were decalcified in 4% EDTA for 2 to 4 weeks before transferring to 70% ethanol. Samples were then paraffin wax embedded and sectioned at 5 µm thickness. Sections were stained with haematoxylin and eosin (H&E; Sigma-Aldrich, Dorset, UK) or toluidine blue (Sigma-Aldrich). Semi-quantitative scoring systems were used to assess cartilage (J.P., H.S.M.) and synovial changes (J.P., T.H.) in each joint by 2 blinded observers as previously described.^20,24^ To assess synovial changes, the intimal hyperplasia, inflammatory infiltrate, subintimal fibrosis and vascularity were all scored from 0 to 3, to give an aggregate score of 0 to 12.^24^ Cartilage changes were assessed using a modified Mankin score (0-100, summed from 0 to 25 per joint quadrant).^20^ The following parameters were scored: cartilage structure (0-10), chondrocyte density (0-4), cell cloning (0-4), metachromasia (interterritorial toluidine blue staining; 0-4) and tidemark/ calcified cartilage/ subchondral bone (0-3). For both cartilage and synovial changes, a higher score indicates a higher degree of tissue abnormality.

Immunofluorescence (IF) was used to determine if hUC-MSCs could be identified in the ovine knee joints, using a mouse monoclonal antibody, MANEM4, clone 6C4, against the epitope peculiar to human emerin which is not present in ovine species (kindly provided by Dr Heidi Fuller [Oswestry]). In brief, all steps were performed at room temperature and sections were washed 3 times in 0.2% Triton X-100 (Sigma-Aldrich) in Phosphate-buffered saline (PBS; Paisley, Life Technologies, UK) between steps unless otherwise stated.^15^ Following deparaffinization and rehydration, antigen retrieval was performed by heating in sodium citrate buffer, pH 6 for 20 minutes at 96 °C. Sections were then blocked for 30 minutes in IF blocking buffer (1% bovine serum albumin/10% horse serum/10% FCS in PBS; Life Technologies). Sections were blotted and incubated with the primary antibody (MANEM4, diluted 1:10 in IF blocking buffer) for 2 hours. Adjacent sections were stained with an isotype-matched IgG1 (Dako, Glostrup, Denmark) as a negative control. Sections were then incubated with the secondary antibody, goat anti-mouse IgG Alexa Fluor 488 (diluted 1:400 in IF blocking buffer) for 1 hour, before washing and mounting the slides in hard-set medium with DAPI (VECTASHIELD^®^, Vector Laboratories, Burlingame, CA). Images were obtained using a Leica SP5 confocal microscope.

### Statistics

Data were tested for normality using the Shapiro-Wilk test, where appropriate. Non-parametric data were analyzed for statistical significance using the Mann–Whitney *U* test and expressed as the median ± interquartile range (IQR). Parametric data were assessed using either the unpaired *t* test with Welch’s correction or the paired *t* test and expressed as the mean ± 95% CI. All data were analyzed using GraphPad Prism 8 (version 8.1.2; San Diego, CA) and deemed statistically significant when *P* < 0.05.

## Results

### Activity Monitoring

**Figure 2.**
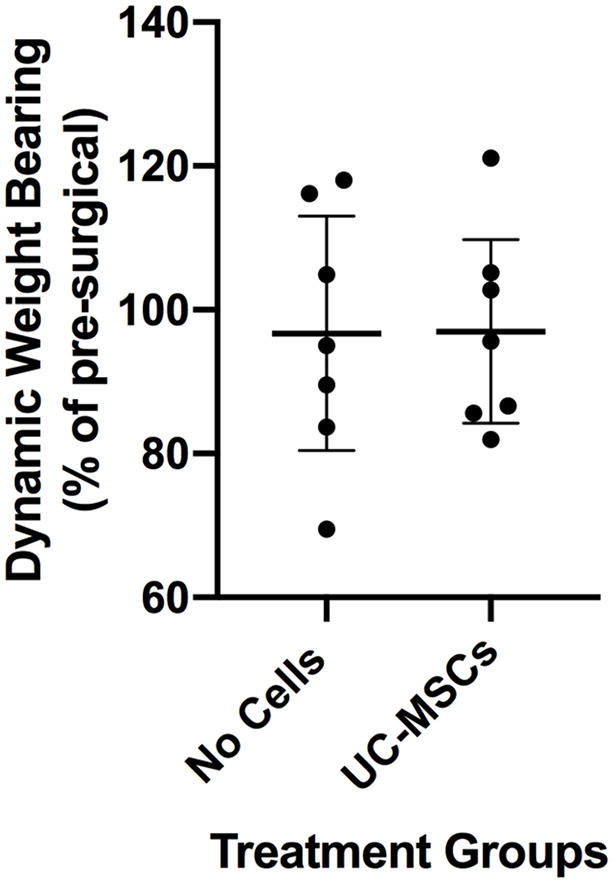
Mean dynamic weight bearing measured as a percentage of the weight bearing after treatment compared with that measured before the surgical injury. Data are presented as the mean ± 95% CI.

The change in mean dynamic weight bearing from before treatment and ~12 weeks after treatment was very similar (*P* = 0.980) between sheep that had received hUC-MSCs compared with the no-cell control group (**Fig. 2**).

### Post-mortem Macroscopic Score

**Figure 3.**
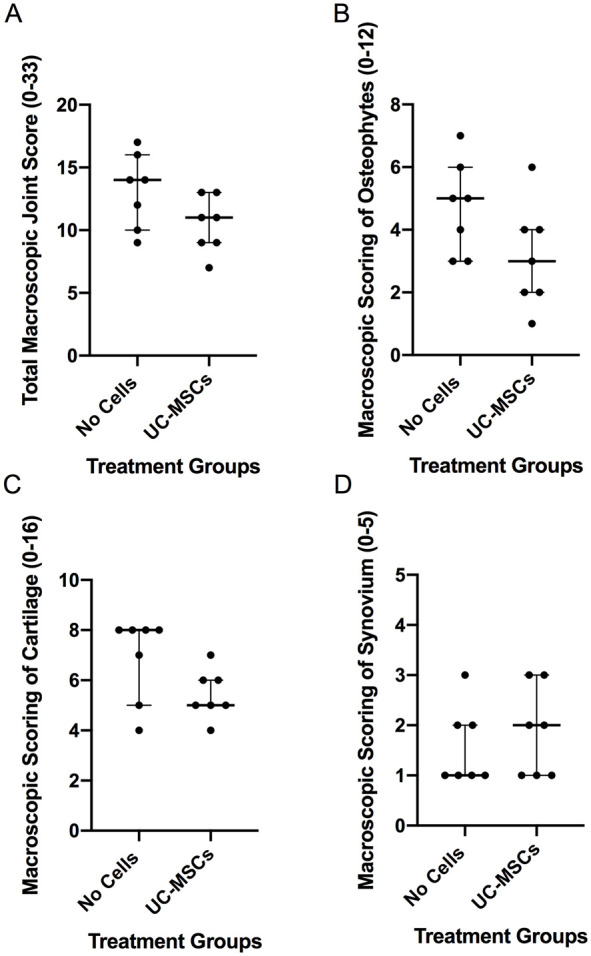
Assessment of macroscopic changes in the treated joints. (**A**) The total macroscopic score (0-33), which incorporates 3 individual parameters: osteophyte development (0-12), gross cartilage damage (0-16) and synovial changes (0-5) showed a lower score in the hUC-MSC treatment group compared with the no cell control group. Similar trends were seen for the individual parameters (**B**) osteophyte development and (**C**) cartilage damage, which were lower in the hUC-MSC treatment group compared with the no cell control, whilst the synovium score (**D**) showed no difference between the 2 treatment groups. Significance was determined below *P* < 0.05. Data are presented as the median ± IQR.

There was an improved (lower) total macroscopic OA joint score in sheep that had received hUC-MSCs (11 ± 4) compared with the no cell control group (14 ± 6), but this did not quite reach significance (*P* = 0.054; **Fig. 3A**; **Table S1**). Assessment of each individual scoring parameter also demonstrated that both parameters, gross articular cartilage damage and osteophyte development, were lower in the hUC-MSCs treatment group compared with the no cell control group, but not significantly (*P* = 0.089 and *P* = 0.096, respectively; **Fig. 3B-C**). There was no significant difference for the macroscopic scoring of the synovium between the 2 treatment groups (*P* = 0.735; **Fig. 3D**).

### Kellgren–Lawrence Score

**Figure 4.**
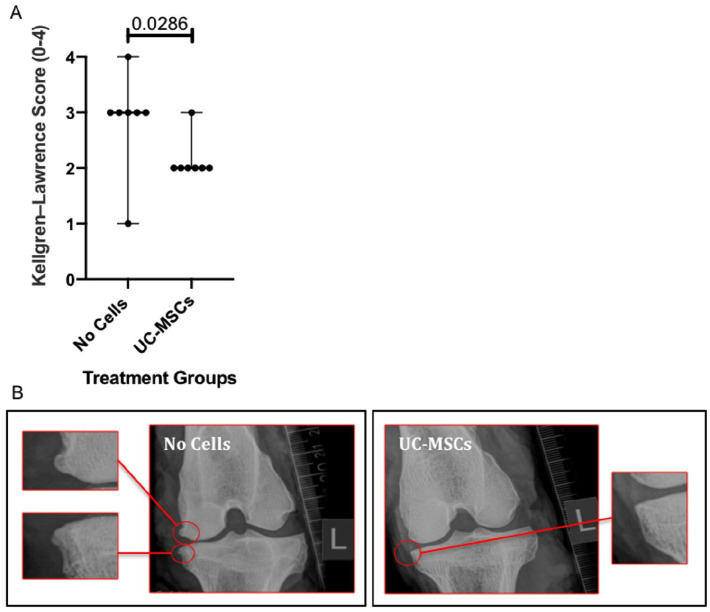
Assessment of radiographic changes. (**A**) Ovine knee joints treated with hUC-MSCs had significantly better (lower) Kellgren–Lawrence scores, compared with the no cell control group. Significance was determined below *P* < 0.05. Data are presented as the median ± IQR. (**B**) Representative x-ray images at 12 weeks post-injury in a joint from the no cells (control) group (left) and one from the hUC-MSCs treatment group (right). The medial side of the joint in the hUC-MSC treated group shows less obvious osteophyte formation (magnified regions) and joint space narrowing compared with that in the no cell control group.

The Kellgren–Lawrence (KL) score, ranging from 0 to 4, showed that joints treated with hUC-MSCs had significantly better (lower) K-L scores (2.0 ± 0) compared with the no cell control group (3.0 ± 0; *P* = 0.028; **Fig. 4**). Typical x-rays from each treatment group are shown in **Figure 4B**.

### Magnetic Resonance Imaging

**Figure 5.**
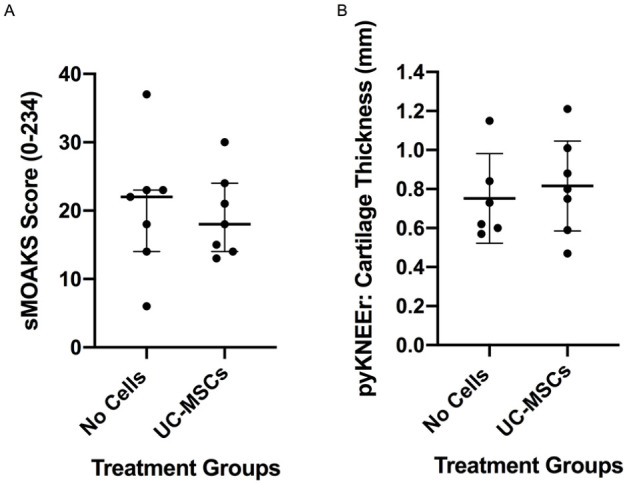
Assessment of joint degeneration on MRI. No significant differences were observed between the 2 treatment groups when assessing osteoarthritic changes using the sheep Magnetic Resonance Osteoarthritis Knee Score (sMOAKS) or cartilage thickness (mm) using pyKNEEr. Significance was determined below *P* < 0.05. Data are presented as the median ± IQR (**A**) and mean ± 95% CI (**B**).

No difference was observed in the sMOAKS score between the hUC-MSC treated group and the no-cell controls (hUC-MSCs 18 ± 10 vs. no cells 22 ± 9; *P* = 0.784; **Fig. 5A**). Likewise, no significant difference in the thickness of the cartilage on the femoral condyles was observed between the treatment group and the no cell control group (hUC-MSCs 0.82 mm, 95% CI [0.585, 1.046]; vs. no cells 0.75 mm, 95% CI [0.522, 0.982]; *P* = 0.632; **Fig. 5B**). In terms of cartilage volume again, no significant differences were observed between the treatment group and the no cell control group (hUC-MSCs 988.7 mm^3^, 95% CI [128.0, 1,849]; vs. no cells 924.8 mm^3^, 95% CI [207.6, 1,642]; *P* = 0.890; data not shown).

### Histological and Immunohistochemical Analyses

**Figure 6.**
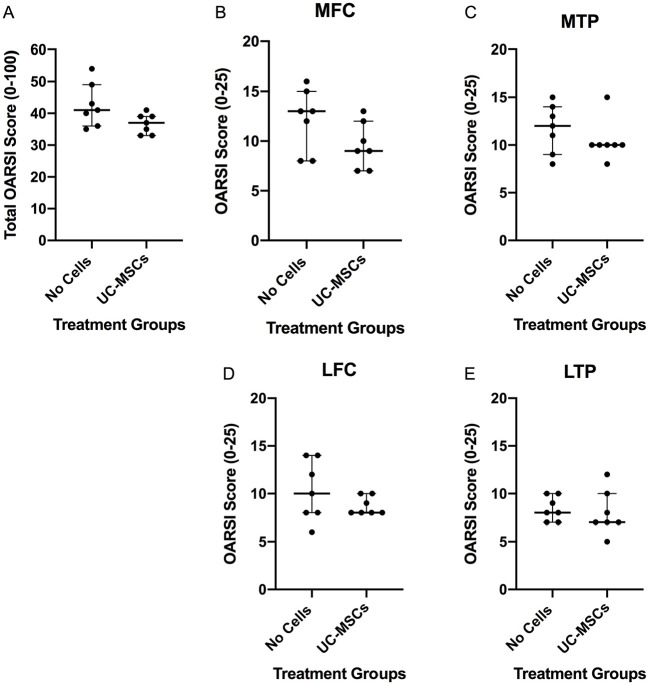
Assessment of microscopic cartilage alterations. (**A**) Histological scoring demonstrated improved (lower) but not significant total microscopic cartilage scores in sheep receiving hUC-MSCs compared with the no cell control group, as well as for each individual joint compartment (**B-E**). Significance was determined below *P* < 0.05. Data are presented as the median ± IQR.

Histological scoring demonstrated improved (lower) microscopic cartilage scores in sheep receiving hUC-MSCs (37 ± 6) compared with the no cell control (41 ± 13), but again, this finding did not quite reach significance (*P* = 0.064; **Fig. 6A**). When assessing each of the joint quadrants individually, again there was no significant difference between the 2 treatment groups (MFC, *P* = 0.142; LFC, *P* = 0.453; MTP, *P* = 0.357; and LTP, *P* = 0.548; **Fig. 6B-E**), although it should be noted that in all cases the median score was lower in the treated group compared with the no cell control and scores were generally more severe on the medial (operated) side of the joint.

**Figure 7.**
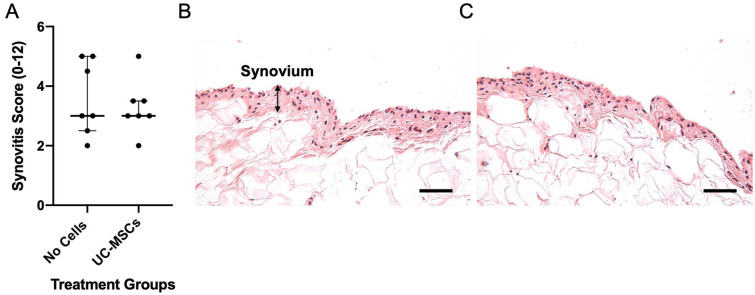
Histological appearance of synovium. Synovitis was assessed using a 12-point system (0 being *normal* to 12 being *severely inflamed*) but was not significantly different between the 2 treatment groups (**A**). Data are presented as the median ± IQR. Representative H&E-stained sections of ovine knee joints 12 weeks post-injury, from a sheep that received the vehicle control, DMEM/F12 (**B**) or hUC-MSCs (**C**). Scale bar = 50 μm.

Furthermore, there was no significant differences in synovitis scores between the 2 treatment groups (hUC-MSCs: 3.0 ± 0.5 vs. no cells 3.0 ± 2.5; *P* = 0.900; **Fig. 7**).

**Figure 8.**
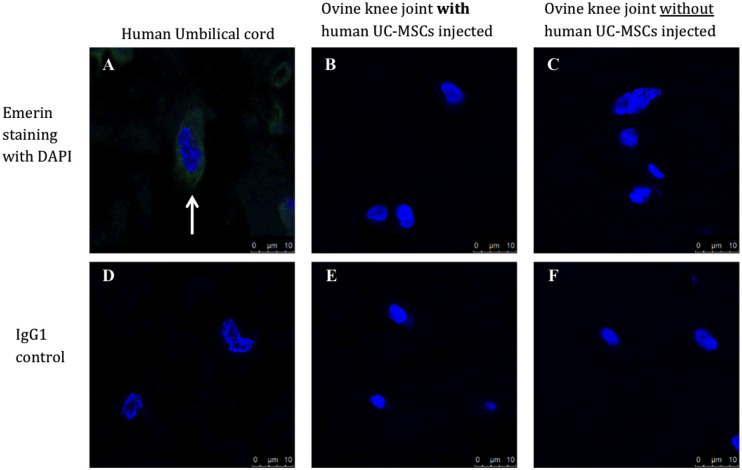
Immunostaining with the human anti-emerin antibody demonstrated (**A**) positive cells (arrows) in a section of human umbilical cord, but not in any of the ovine knee joints (**B, C**), including those which had hUC-MSCs (**B**) applied. All isotype matched controls showed negative staining (**D-F**). Sections were counterstained with DAPI (blue nuclei).

No human cells were detected by immunostaining with the antibody to human emerin in paraffin wax-embedded sections of the joints from sheep receiving human UC-MSCs 8 weeks post-implantation (**Fig. 8**). They were, however, clearly visible in sections of human umbilical cord, used as a positive control tissue.

## Discussion

Extensive work has been undertaken to evaluate the characteristics of hUC-MSCs and also their effect in murine preclinical models of OA.^15,16^ This study of hUC-MSCs in a model of early to moderate OA in a large animal is the next logical progression in the translational pipeline for developing hUC-MSCs as a treatment for human patients with OA.

The hUC-MSCs were obtained via a “hybrid” process of growing hUC-MSCs, with culture expansion, initially in monolayer, followed by use of the Quantum^®^ bioreactor system. Such up-scale expansion can provide a cost-effective method of producing large numbers of cell doses whilst complying with GMP-regulations.^25^ Previous work in our group has shown little difference with regard to MSC characterization, tri-lineage differentiation or telomere length, between hUC-MSCs grown using this bioreactor compared with traditional tissue culture plastic protocols.^9^ In addition, there were very high levels of gene expression for the immunomodulatory molecule, indoleamine 2, 3-dioxygenase (IDO), by Quantum^®^ generated hUC-MSCs when stimulated with interferon gamma, thus warranting further *in vivo* investigation of Quantum^®^ expanded hUC-MSCs potency.

In order for cell therapy to be considered a useful and cost-effective treatment for OA, it should alter the disease progression and pathogenesis, so by necessity be applied earlier in the OA spectrum than at the end-stage when joint prostheses are used, perhaps even being applied at the pre-OA stage.^26^ Sheep are considered a suitable large animal model of musculoskeletal tissues, since the biomechanics of the ovine stifle joint is similar to the human knee^20,27^ and it is highly translatable to the human clinical setting.^28^ Meniscal release procedures, such as has been used in this study, result in a mild OA with moderate changes within 12 weeks,^17,18^ thus allowing the application of a treatment modality part way through the pathogenesis of OA.

As hypothesized, the hUC-MSCs appeared to delay the progression of OA, with significantly lower KL scores in the treatment arm compared with the no-cell control group. Likewise, the macroscopic and histological scoring showed better scores following treatment with hUC-MSCs, although this did not quite reach significance. This suggests that perhaps the study was underpowered and indeed the numbers in each group were fairly small. However, this number had been calculated to be adequate from an earlier study based on histology as an outcome measure to achieve a 0.8 level of power (personal information; F Henson, Cambridge). Furthermore, when looking at the synovitis scores, a non-significant difference between the 2 treatment groups suggests that the hUC-MSCs did not evoke an inflammatory response in any of the treated animals, at least at the time-point measured.

The chronology of OA development may also in part be responsible for a lack of significance in some parameters, for example, in gait analysis. Cake *et al* *.*^17^ found vertical ground reaction forces varied considerably with time post-meniscal injury compared with baseline, with maximal reduction of peak forces at 2.5 and 8 weeks but a tendency to return towards normal by 12 weeks.^17^ Similarly, the dynamic weight bearing in sheep which had undergone the same meniscal destabilization as in the current study, showed an improvement after 3 weeks when treated with 120 µg of a GDF-5 analogue compared with untreated controls, but this difference between treated and controls decreased with time to 11 weeks and, as in the current study, did not reach significance.^19^ Indeed Newell *et al.*^18^ showed there to be a correlation between activity and the severity of pathology. With several outcome measures showing a trend to improvement in pathological OA features but not reaching significance, perhaps larger sample numbers and extending the timescale to 24 or 26 weeks may have rendered a higher level of significance. Furthermore, the continual monitoring of the sheep using smart technology, such as commercially available telemetric systems, for example, Fitbark, which measures movement behavior, may have allowed more subtle changes to be monitored over time.^18^ In addition, to gain more information on the mode of action (MoA) of the implanted cells, studying changes in the inflammatory status of different tissues such as the meniscus, particularly at different time-points, may provide useful and critical information.

Two of the treated sheep demonstrated a local reaction the day after injection. Initial inflammation as an adverse reaction is reported by one other group using an intra-articular injection of xenogeneic cell products. Punzón *et al.*^29^ found an initial increase in pain and lameness in 4 of the 40 dogs which were treated with equine UC-MSCs in their dog OA study. As in our study, they treated it with anti-inflammatory drugs and suggest that it did not influence the long-term benefit of the cells.

The MoA of MSCs, including those derived from UCs, remains uncertain. Whilst the previously held belief was that MSCs would differentiate into the cell type of the tissue undergoing repair, depending on local cues (e.g., into chondrocytes for cartilage injuries), this is no longer thought to be the main MoA.^30^ Rather, MSCs do appear to be responsive to the host environment, but with a paracrine response such that if inflammation is present they may be stimulated to synthesize anti-inflammatory molecules and so diminish or dampen it down. For example, previous in vitro studies in our center have shown that hUC-MSCs upregulate IDO gene expression after exposure to a pro-inflammatory stimulus (IFN-γ).^9^ IDO, a potent immunomodulatory molecule, appears to deplete tryptophan via the kynurenine pathway causing the suppression of T-cells, minimizing the local inflammatory response.^31^ In addition, they may also modulate the host’s cells to mount a therapeutic response.^30^ Long-term retention of viable MSCs may not be necessary for this, with many studies finding as we did, that implanted MSCs were not engrafted into the treated tissues, with few or no cells possible to identify soon after implantation.^32^ This finding is not universal, however, and some studies are able to identify implanted cells some time post-implantation. For example, labeled autologous MSCs which had been implanted into intervertebral disks were detected in 75% of those disks 8 months later.^33^ Similarly, in a study of Hartley-Dunkin guinea pigs with naturally occurring OA, MSCs which had been injected intra-articularly with hyaluronan, could be identified at 5 weeks post-implantation, the latest time-point in that study.^34^

Umbilical cord cells, prepared in different ways, are progressing along the translational pathway to the clinic as appropriate cells for allogeneic cell therapies in several fields. ORBCELL™ (Orbsen Therapeutics, Ireland), which are CD362-enriched hUC-MSCs, are being used in the United Kingdom in a phase I/II trial of Acute Respiratory Distress Syndrome (REALIST^35^; and also in a phase II trial of autoimmune hepatitis, http://clinicaltrials.gov: NCT02585622). In Vietnam, Vinmec are running a phase I/II trial of UC-MSCs in chronic obstructive pulmonary diseases (COPD; https://clinicaltrials.gov: NCT04433104). In the veterinary world equine UC-MSCs already have approval for use in the treatment of OA in dogs^29^ and horses.^36^ Their product, HorStem^®^, received a marketing authorization by the European Medicines Authority (EMA) in 2019.^36^ The study leading to this approval describes how of 33 horses with mild to moderate OA, 16 received an injection of HorStem^®^ into the affected joint and 17 horses received a placebo injection.^36^ They were examined for lameness (on a scale of 0 to 5, where 0 is *normal*) at days 14, 35, and 63. Treatment was considered successful if the lameness reduced to 0 or 1. There was a success rate of 75% in the cell treated group at day 63, compared with 25% in the placebo group.^36^

The results of the present study provide further support to the premise that hUC-MSCs could provide a suitable allogeneic therapy for treating OA in humans in the early stages of the disease. Even without knowing the exact MoA of the cells, one cannot ignore the mounting evidence indicating the ability of MSCs to alter the joint environment and appear to stimulate regeneration in some tissues^37^ and retard destruction of other tissues.^38^ In conclusion, we suggest that a phase I/II clinical trial of hUC-MSCs for treating early OA in humans is warranted, whilst also addressing the outstanding challenge of identifying clear potency markers.^30^

## Supplemental Material

sj-docx-1-car-10.1177_19476035241287832 – Supplemental material for Intra-Articular Injection of Human Umbilical Cord-Derived Mesenchymal Stromal Cells Reduces Radiographic Osteoarthritis in an Ovine Model Supplemental material, sj-docx-1-car-10.1177_19476035241287832 for Intra-Articular Injection of Human Umbilical Cord-Derived Mesenchymal Stromal Cells Reduces Radiographic Osteoarthritis in an Ovine Model by Jade Perry, Claire Mennan, Paul Cool, Helen S. McCarthy, Karin Newell, Timothy Hopkins, Charlotte Hulme, Karina T. Wright, Frances M.D. Henson and Sally Roberts in CARTILAGE
