# Supplementary material for: Intra-Articular Injection of Human Umbilical Cord-Derived Mesenchymal Stromal Cells Reduces Radiographic Osteoarthritis in an Ovine Model
Source: Cartilage. 2024 Nov 3;17(3):684–94. doi: 10.1177/19476035241287832 (PMC11556672; doi:10.1177/19476035241287832)
Supplement: Supplementary file 1 [file sj-docx-1-car-10.1177_19476035241287832.docx]

**Supplementary Data**

**Table 1.** Scoring parameters and results for each treatment group (median ± IQR or *mean ± 95% CI*; n=7 joints per group).

| **Scoring System** | **Cell (hUC-MSC) treated group** | **Vehicle Control group** | **p value** | **Range of Scoring system**  (low = normal, high = OA) | **Reference** |
| --- | --- | --- | --- | --- | --- |
| *Activity Monitoring* | 96.98, 95% CI 84.21, 109.8 | 96.71, 95% CI 80.41, 113 | p=0.980 | Continuous Data | Gigout *et al,* 2022. |
| Macroscopy Score | 11±4 | 14±6 | p=0.054 | 0-33 | Little *et al,* 2010. |
| Kellgren Lawrence OA Score | 2.0±0 | 3.0±0 | p=0.028 | 0-4 | Kellgren & Lawrence 1957. |
| sMOAKS Score  (MRI) | 18±10 | 22±9 | p=0.784 | 0-234 | Moya-Angeler *et al*, 2016. |
| *Cartilage thickness; mm (MRI; pyKNEEr)* | 0.82mm, 95% CI 0.585, 1.046 | 0.75mm, 95% CI 0.522, 0.982 | p=0.632 | Continuous Data | Boranetti *et al*, 2020. |
| Modified Mankin Score  (OARSI histopathology initiative) | 37±6 | 41±13 | p=0.064 | 0-100 | Little *et al,* 2010. |
| Synovitis Score | 3.0±0.5 | 3.0±2.5 | p=0.900 | 0-12 | Cake *et al*, 2008. |
